# Arsenic in Sulphuric Acid

**Published:** 1847-06

**Authors:** 


*Arsenic in Sulphuric Acid.*—M. Dupasquier recommends sulphate of barium as a convenient and effectual substance to free sulphuric acid from the arsenic it so frequently contains. M. Erdman says it has been long employed in the Hertz, where the proportion of arsenic is very great, and that the arsenic is rendered perfectly pure.—*Ibid, from Jour. f. Prakt. Chem.*

